# Malnutrition in hospitalized adults in the United States, 2016–2019

**DOI:** 10.1002/jhm.13456

**Published:** 2024-07-09

**Authors:** Ajay Bhasin, Lynn Huang, Meng‐Shoiu Shieh, Penelope Pekow, Peter K. Lindenauer, Tara Lagu

**Affiliations:** ^1^ Department of Medicine, Division of Hospital Medicine Northwestern University Feinberg School of Medicine Chicago Illinois USA; ^2^ Department of Pediatrics, Division of Hospital‐Based Medicine Northwestern University Feinberg School of Medicine Chicago Illinois USA; ^3^ Department of Preventive Medicine Northwestern University Feinberg School of Medicine Chicago Illinois USA; ^4^ Department Center for Health Services and Outcomes Research Institute for Public Health and Medicine, Northwestern University Feinberg School of Medicine Chicago Illinois USA; ^5^ Department of Healthcare Delivery and Population Sciences University of Massachusetts Chan Medical School—Baystate Springfield Massachusetts USA; ^6^ Department of Medicine University of Massachusetts Chan Medical School—Baystate Springfield Massachusetts USA

## Abstract

**Background:**

Malnutrition in hospitalized patients is associated increased length of stay, cost, readmission, and death. No recent studies have examined trends in prevalence or outcomes of hospitalized patients with a diagnosis of malnutrition.

**Objectives:**

To study the prevalence of malnutrition diagnostic codes and associated hospital outcomes in the United States between 2016 and 2019.

**Methods:**

We conducted a retrospective trends study to identify use of malnutrition codes in hospitalizations in the National Inpatient Sample between 2016 and 2019. We used direct standardization by logistic regression to adjust outcomes of percutaneous gastrostomy tube placement, mechanical ventilation, and death for age, Gagne comorbidity score, and sex. We then used linear regression to test for trends over time by malnutrition type.

**Results:**

Across all hospitalizations, codes for diagnoses of nonsevere malnutrition and severe malnutrition were present in 3.7% and 4.1% of hospitalizations, respectively. Codes for any malnutrition increased over time, from 6.6% in 2016 to 8.6% in 2018 (*p* = .03). Codes for severe malnutrition increased from 3.3% to 4.7% (*p* = .01). Among hospitalizations with coded severe malnutrition diagnoses, there was a statistically significant decrease in adjusted rate of death over time (−0.54% per year, *p* = .03) which was not seen in hospitalizations without coded malnutrition diagnoses.

**Conclusions:**

Use of malnutrition diagnosis codes increased significantly from 2016 to 2019. During this time, mortality among hospitalizations with a diagnosis code for severe malnutrition decreased. Though the increased prevalence of malnutrition codes may represent a change in the clinical characteristics of hospitalized patients, the decline in mortality suggests some of the increase may be due to lower threshold for coding and assignment of the diagnosis to less ill patients.

## INTRODUCTION

Malnutrition develops when the body is deprived of vitamins, minerals, and other nutrients (such as proteins, carbohydrates, and fats) required to maintain normal bodily function.^1^ Severe malnutrition, an extreme form of malnutrition, is categorized by low body mass index and cachexia, among other findings.^2^ In the absence of anorexia nervosa or starvation, severe malnutrition is generally not a primary disorder but is associated with other chronic conditions (i.e., disease‐associated malnutrition), some of which are not curable (e.g., cancer).^3^ Current methods used to identify patients with malnutrition include the Academy of Nutrition and Dietetics/American Society for Parenteral and Enteral Nutrition (AND/ASPEN) criteria and Global Leadership Initiative on Malnutrition (GLIM) criteria. These criteria are different from each other, subjective, and can be difficult for nonexperts to use.^4^

In hospitalized patients, identification of malnutrition as a comorbidity provides information about the risk for hospital complications, such as poor wound healing, increased length of stay and cost, readmission, or death.^2^ Hospitals are incentivized to diagnose malnutrition by the Inpatient Prospective Payment System, which uses Medicare Severity Diagnosis‐Related Groups to identify a “payment weight.” When severe malnutrition is included on a patient's diagnosis list, a major complication or comorbidity (MCC) classifier is almost always added to the hospitalization claim.^5^ Adding an MCC classifier increases reimbursement and modifies risk adjustment for quality measures relative to diagnoses without a MCC classifier.^5^ Given the increased costs and worse outcomes associated with caring for patients with severe malnutrition, hospitals have an incentive to accurately diagnose and document the presence of malnutrition.

Thus, there are several reasons the prevalence of malnutrition in hospitalized patients may have changed over time: complicated and varied criteria for diagnosis, incentives for coding these conditions, and an association with worse outcomes, which may indicate higher patient complexity. There are no recent studies that have examined the change in prevalence of malnutrition or evaluated associations with illness severity, treatment for malnutrition, or death. We aimed to examine recent, short‐term trends in the epidemiology of malnutrition among hospitalized patients in the United States to understand how use of this diagnosis has changed over time.

## METHODS

We used the National Inpatient Sample (NIS) to conduct a retrospective trends study of hospitalizations in the United States from 2016 until 2019.^6^ The NIS is a deidentified administrative database published by the Agency for Healthcare Quality and Research (AHRQ). It is constructed from a 20% sample of inpatient hospitalizations nationally, gathered from state inpatient databases. It is comprised of International Classification of Disease (ICD) diagnosis and procedure codes, patient level characteristics including demographics, and hospitalization characteristics including length of stay and charges. We chose the years 2016–2019 because of the consistent presence of ICD‐10 diagnoses for malnutrition (there was not 1:1 conversion of ICD‐9 diagnosis codes for malnutrition to ICD‐10 for years before 2016). We did not include the year 2020, because it was not available at the time of analysis and because the data set would have been influenced by the coronavirus disease 2019 (COVID‐19) pandemic.

We excluded elective, surgical, or obstetric hospitalizations and limited the sample to patients aged 18 years or older. We then identified baseline demographic characteristics including age, which was split into quartiles, sex, zip code income quartile, and race and ethnicity, which was split into four categories: White, Black, Hispanic, and other (as defined by the NIS). We categorized presence of comorbidities using the Gagne index,^7^ an integer score describing patient mortality risk. We calculated the Gagne score for each patient using ICD‐10 codes (Supporting Information S1: Appendix A) and then split the index into quartiles of comorbidity burden. We identified diagnoses of malnutrition, moderate malnutrition, severe malnutrition, and other malnutrition types by presence of ICD‐10 codes for each, respectively (Supporting Information S1: Appendix A), in any diagnostic position. When multiple malnutrition codes were present for a hospitalization, we categorized the most severe malnutrition type. We combined mild malnutrition, moderate malnutrition, and other malnutrition diagnosis codes into a single “nonsevere” malnutrition category.

To better understand the severity of illness, outcomes, and treatments in patients without a diagnosis of malnutrition, we collected information on mechanical ventilation, death, and percutaneous gastrostomy (PEG) tube placement by ICD‐10 procedure codes (Supporting Information S1: Appendix A). We chose mechanical ventilation because it is a marker of higher illness severity, and, in general, patients who are critically ill are at high risk for being malnourished. We included PEG tube placement because it suggests an attempt to treat malnutrition was made during the hospitalization, which would be consistent with very severe cases (although PEG placement is limited to patients who can tolerate enteral feeding).

We used counts and percents to describe demographics and categorical variables. As age and Gagne score were not normally distributed, we used median and interquartile range (IQR) for these variables.

For large sample size populations, standard statistical testing (e.g., *χ* ^2^ testing) often generates statistically significant differences for most variables due to narrow confidence intervals. To avoid this and to identify variables with meaningful differences between groups, we compared characteristics between hospitalizations with malnutrition codes and no malnutrition codes using absolute standardized mean differences (ASD). In general, an ASD of 10% or greater is considered clinically important.^8^ We used direct standardization by logistic regression to adjust rates of mechanical ventilation, PEG tube placement, and death by age group, Gagne score quartile, and gender per each malnutrition diagnosis category (severe, nonsevere, or no malnutrition).^9^ We used linear regression to test for trend over time to evaluate changes in standardized rates per year. We used SAS (9.4.2) software with appropriate survey weighting as suggested by the AHRQ for statistical analysis. The full NIS sample is reconstructed by applying discharge weights provided by the AHRQ using survey packages and software to unweighted NIS data to generate national‐level estimates.^10^ This study was deemed “not human subjects research” by the Northwestern University Institutional Review Board.

## RESULTS

**Figure 1:**
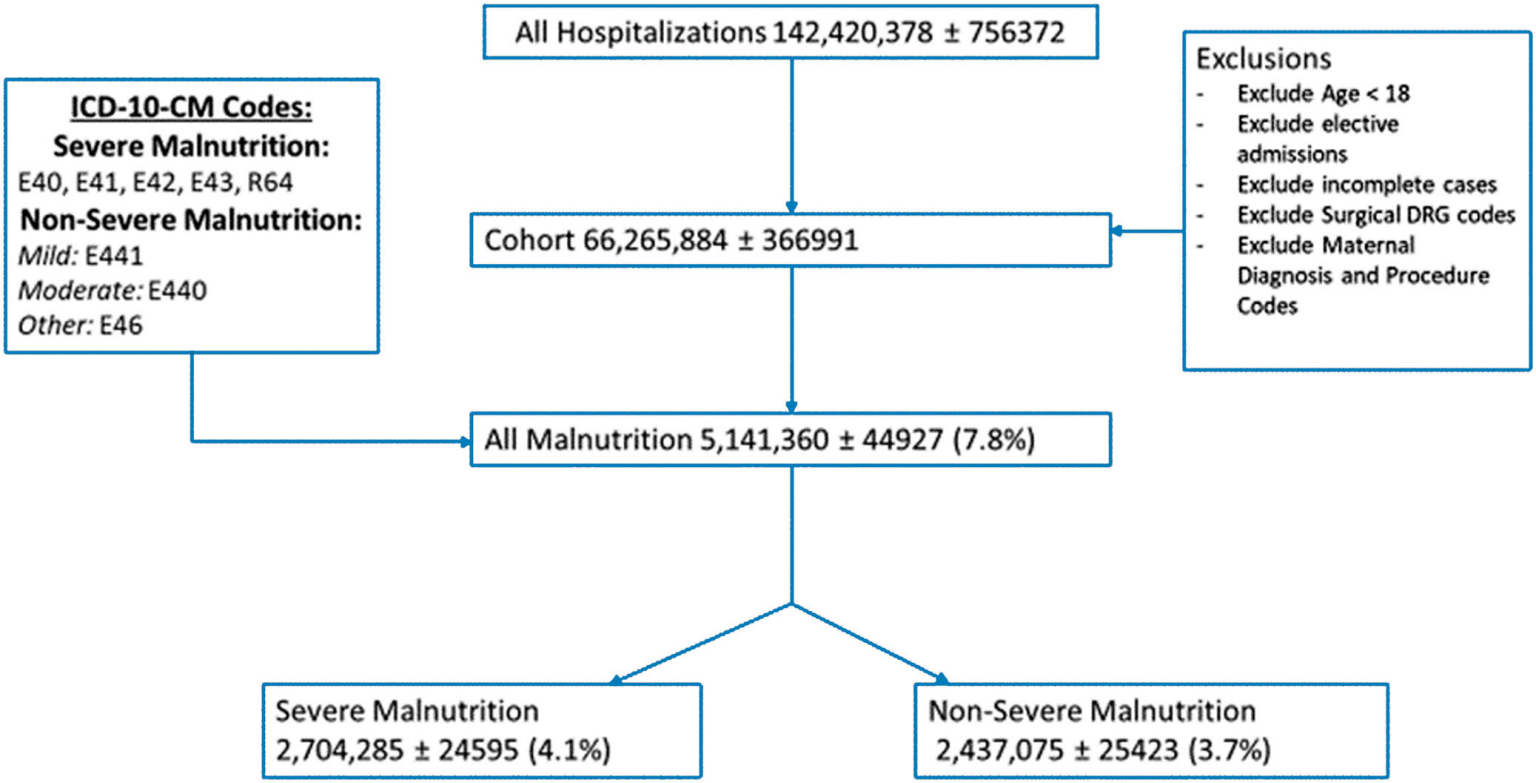
Cohort Identification flowchart.

From 2016 to 2019, we identified more than 142 million hospitalizations in the United States (Figure 1). Of these, 66,265,884 ± 366,991 hospitalizations met our inclusion criteria (Table 1 and Figure 1), and 2,437,075 ± 25,423 (3.7%) of these hospitalizations contained a diagnosis code for nonsevere malnutrition, whereas 2,704,285 ± 24,595 (4.1%) hospitalizations contained a code for severe malnutrition. Hospitalized patients with codes for nonsevere malnutrition diagnoses (median 70 years, IQR [58, 81]) and codes for severe malnutrition diagnoses (70 years, IQR [59, 81]) tended to be older than those without codes for malnutrition diagnoses (63 years, IQR [49, 77]). The Gagne combined comorbidity score was higher in those with codes for nonsevere (4.8, IQR [3.2, 6.9]) and severe (5.0, IQR [3.4, 7.3]) malnutrition diagnoses relative to those without codes for malnutrition diagnoses (1.5, IQR [0.1, 3.4]). Unadjusted rates of mechanical ventilation were higher in patients with codes for nonsevere (7.0%, ASD 0.16) and severe (7.1%, ASD 0.17) malnutrition diagnoses than those without codes for malnutrition diagnoses (3.3%). Unadjusted rates of death were higher in those with codes for nonsevere (5.5%, ASD 0.17) and severe (9.2%, ASD 0.30) malnutrition diagnoses than without codes for malnutrition diagnoses (2.3%), respectively.

**Table 1:** Demographic characteristics and outcomes of patients by type of malnutrition diagnosis, 2016–2019.

	Overall		No malnutrition		Nonsevere malnutrition			Severe malnutrition		
	***n* (SE)**	**%**	***n* (SE)**	**%**	***n* (SE)**	**%**	**ASD** a	***n* (SE)**	**%**	**ASD** a
Total hospitalizations	66,265,884 (366,991)	100	61,124,524 (338,573)	92.2	2,437,075 (25,423)	3.7	–	2,704,285 (24,595)	4.1	–
Age (median, [IQR])	64 [50, 77]	–	63 [49, 77]	–	70 [58, 81]	–	0.331	70 [59, 81]	–	0.358
Gender										
Male	32,018,209 (180,210)	48.3	29,558,290 (166,577)	48.4	1,140,135 (11,994)	46.8	3.2	1,319,865 (12,372)	48.8	0.8
Female	34,247,593 (190,159)	51.7	31,566,233 (175,211)	51.6	1,296,940 (13,782)	53.2	3.2	1,384,420 (12,666)	51.2	0.8
Race/ethnicity										
White	43,825,198 (297,768)	66.1	40,401,453 (274,562)	66.1	1,643,635 (19,148)	67.4	2.75	1,780,110 (17,709)	65.8	0.63
Black	10,724,461 (132,687)	16.2	9,883,946 (121,424)	16.2	376,580 (7515)	15.5	2.68	463,935 (8072)	17.2	1.92
Hispanic	6,360,717 (105,686)	9.6	5,942,237 (99,466)	9.7	206,470 (4499)	8.5	4.17	212,010 (4542)	7.8	6.73
Other	5,355,508 (103,485)	8.1	4,896,888 (94,650)	8	210,390 (5104)	8.6	2.17	248,230 (6009)	8.1	0.37
Zip income quartile										
0%–25%	21,154,325 (200,393)	31.9	19,527,600 (185,205)	31.9	786,930 (14,743)	32.3	0.86	839,975 (11,069)	31.1	1.72
25%–50%	17,438,639 (145,591)	26.3	16,124,799 (135,230)	26.4	627,660 (7723)	25.8	1.37	686,180 (7947)	25.4	2.28
50%–75%	15,417,976 (140,119)	23.3	14,214,406 (129,743)	23.3	568,805 (7189)	23.3	0	634,765 (7679)	23.5	0.47
75+%	12,254,943 (171,161)	18.5	11,257,718 (157,578)	18.4	453,680 (7900)	18.6	0.52	543,545 (9876)	20.1	4.31
Gagne score (median, [IQR])	1.7 [0.3, 3.8]	–	1.5 [0.1, 3.4]	–	4.8 [3.2, 6.9]	–	1.33	5.0 [3.4, 7.3]	–	1.37
Peg tube placement^b^	312,260 (2980)	0.47	157,045 (1796)	0.26	65,670 (879)	2.7	20.31	89,545 (1143)	23.3	23.14
Mechanical ventilation^b^	2,409,035 (17,139)	3.6	2,045,555 (14,885)	3.3	170,835 (2191)	7	16.18	192,645 (2154)	7.1	17.18
Death^b^	1,815,534 (13,013)	2.7	1,432,025 (10,494)	2.3	133,885 (1550)	5.5	16.59	249,625 (2566)	9.2	29.97

Abbreviations: ASD, absolute standardized mean difference; IQR, interquartile range; SE, standard error.

^a^ ASD from no malnutrition.

b Denotes category with ASD > 10% from reference category (no malnutrition).

The proportion of hospitalizations with a diagnosis code for malnutrition increased between 2016 and 2019 (6.6%–8.6%, *p* = .03, Table 2). The prevalence of codes for severe malnutrition diagnoses (3.3%–4.7%, *p* = .01) increased more than nonsevere malnutrition diagnoses (3.4%–3.8%, *p* = .11), but both increased overall (Table 2).

**Table 2:** Malnutrition hospitalizations and unadjusted outcomes by year.

	2016		2017		2018		2019			
	*n* (SE)	%	*n* (SE)	%	*n* (SE)	%	*n* (SE)	%	Slope	*p*
All hospitalizations	16,104,991 (151,383)	–	16,642,934(158,882)	–	16,754,264 (159,836)	–	16,763,695 (164,807)	–	–	–
Deaths	451,405 (5524)	2.8	456,235 (5698)	2.74	452,105 (5601)	2.7	455,790 (5978)	2.72	−0.028	.163
Peg tube use	78,900 (1405)	0.49	78,865 (1376)	0.47	76,805 (1324)	0.46	77,690 (1381)	0.46	−0.01	.0871
Mechanical ventilation	602,675 (7701)	3.74	576,240 (7246)	3.46	594,500 (7408)	3.55	616,760 (7844)	3.68	−0.009	.908
Length of stay (LOS)	4.64 (0.02)	–	4.62 (0.02)	–	4.64 (0.02)	–	4.69 (0.02)	–	0.017	.265
Malnutrition hospitalizations	1,069,600 (17552)	6.64	1,273,385 (20943)	7.65	1,365,160 (22113)	8.15	1,433,215 (23343)	8.55	0.62	.0252
Severe malnutrition	528,500 (9116)	3.28	654,145 (11175)	3.93	730,355 (12066)	4.36	791,285 (13246)	4.72	0.475	.0098
Deaths	53,035 (1086)	10.04	62,110 (1205)	9.49	65,055 (1205)	8.9	69,425 (1285)	8.77	−0.44	.028
Peg tube use	17,995 (453.6)	3.4	21,905 (539.7)	3.35	23,825 (553.0)	3.26	25,820 (632.3)	3.26	−0.05	.052
Mechanical ventilation	42,015 (961.9)	7.95	46,625 (1008)	7.13	49,605 (1030)	6.79	54,400 (1117)	6.87	−0.36	.128
LOS: severe malnutrition	7.67 (0.06)	–	7.54 (0.05)	–	7.51 (0.05)	–	7.58 (0.05)	–	−0.03	.443
Nonsevere malnutrition	541,100 (10497)	3.36	619,240 (12421)	3.72	634,805 (13174)	3.79	641,930 (13375)	3.83	0.148	.111
Deaths	33,100 (721.3)	6.12	34,340 (746.9)	5.55	33,045 (753.2)	5.21	33,400 (764.7)	5.2	−0.26	.073
Peg tube use	17,415 (448.0)	3.22	16,725 (433.8)	2.7	16,020 (417.2)	2.52	15,510 (404.6)	2.42	−0.41	.065
Mechanical ventilation	43,190 (1113)	7.98	42,770 (1029)	6.91	41,830 (1051)	6.59	43,045 (1087)	6.71	−0.05	.161
LOS: nonsevere malnutrition	7.44 (0.07)	–	7.14 (0.06)	–	7.12 (0.06)	–	7.27 (0.06)	–	−0.053	.536
No malnutrition	15,035,392 (136,204)	93.36	15,369,549 (138,939)	92.35	15,389,103 (140,291)	91.85	15,330,479 (143,700)	91.45	−0.62	.03
Deaths	365,270 (4493)	2.43	359,785 (4592)	2.34	354,005 (4572)	2.3	352,965 (4895)	2.3	−0.04	.09
Peg tube use	43,490 (920.9)	0.29	40,235 (850.4)	0.26	36,960 (771.2)	0.24	36,360 (787.2)	0.24	−0.02	.07
Mechanical ventilation	517,470 (6687)	3.44	505,705 (6548)	3.29	503,065 (6454)	3.27	519,315 (6842)	3.39	−0.02	.73
LOS: nonsevere malnutrition	4.43 (0.02)	–	4.40 (0.02)	–	4.40 (0.02)	–	4.43 (0.02)	–	0	1

Abbreviation: SE, standard deviation.

**Table 3:** Adjusted rates of outcomes over time by malnutrition type.

	2016 (%)	2017 (%)	2018 (%)	2019 (%)	Slope	*p*
Severe malnutrition						
Death	12.2	11.4	10.8	10.6	−0.54	.03
Peg tube	3.22	3.27	3.19	3.13	−0.04	.23
Invasive mechanical ventilation	9.16	8.31	7.91	7.91	−0.42	.09
Nonsevere malnutrition						
Death	7.62	6.82	6.41	6.48	−0.38	.11
Peg tube	3.11	2.65	2.25	2.34	−0.27	.10
Invasive mechanical ventilation	9.56	8.22	7.89	8.10	−0.47	.20
No malnutrition						
Death	1.85	1.79	1.77	1.82	−0.01	.59
Peg tube	0.25	0.23	0.22	0.21	−0.01	.02
Invasive mechanical ventilation	3.29	3.14	3.14	3.23	−0.02	.68

Abbreviation: SE, standard error.

* Adjusted for age group, Gagne score quartile, and gender per malnutrition diagnosis category.

Among the cohort of patients with diagnosis codes for severe malnutrition, we observed a decline in the adjusted death rate from 12.2% to 10.6% (−0.54% per year, *p* = .03). We observed a similar trend in rates of invasive mechanical ventilation (−0.42% per year, *p* = .09) that did not reach statistical significance. There was no change in receipt of PEG‐tube (−0.04% per year, *p* = .23) (Table 3).

**Figure 2:**
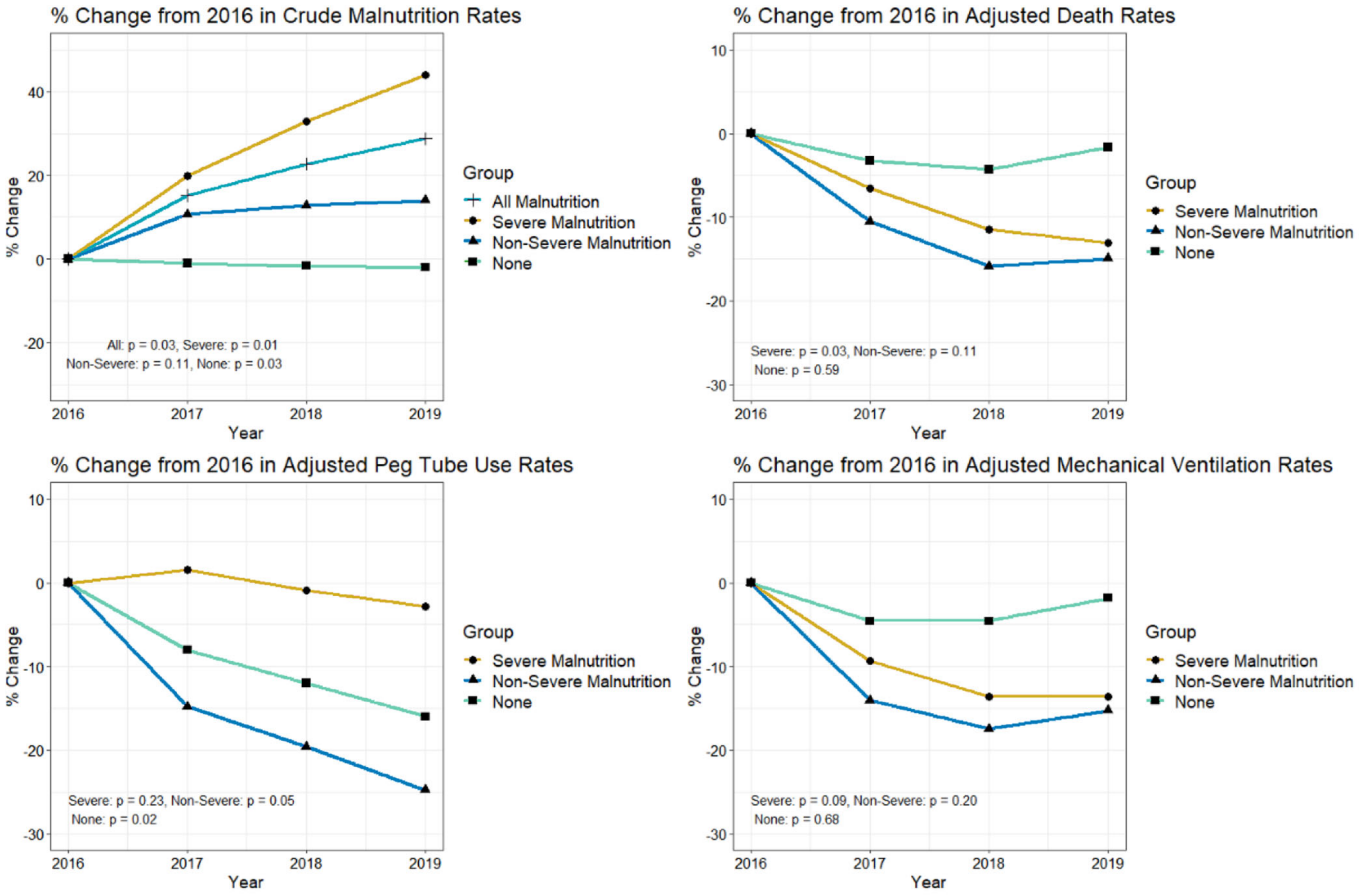
From left to right, then top to bottom: (1) % change in crude rates of malnutrition diagnoses from 2016. (2) % change in adjusted death rates among malnutrition groups from 2016. (3) % change in adjusted PEG tube use rates among malnutrition groups from 2016. (4) % change in adjusted invasive mechanical ventilation rates among malnutrition groups from 2016. *Analyses for Panel 2, 3, 4 were adjusted for age group, Gagne score quartile, and gender per malnutrition diagnosis category.

Adjusted rates for mortality, PEG‐tube placement, and mechanical ventilation also decreased in those with codes for nonsevere malnutrition diagnoses (−0.38% per year [*p* = .11], −0.27% per year [*p* = .10], and −0.47% per year [*p* = .20], respectively), but the trends were not statistically significant. In those without codes for malnutrition, the adjusted rates did not change. Figure 2 depicts the trends as a percent change relative to the 2016 baseline prevalence for crude rates of malnutrition and adjusted outcomes by malnutrition type.

## DISCUSSION

Malnutrition is known to be associated with poorer outcomes including increased length of stay, higher cost of care, and increased mortality. In this study of more than 66 million US hospitalizations, we observed a 50% increase in the use of diagnosis codes for malnutrition between 2016 and 2019. The greatest change was in the proportion of patients who were assigned a diagnosis code of severe malnutrition. Over the same period, we found mortality among patients with severe malnutrition diagnoses declined.

The published literature suggests 20%–50% of inpatients meet clinical criteria for a diagnosis of malnutrition.^3^ We could find no recent studies describing use of diagnosis codes for malnutrition in hospitalized adults in the United States, but our finding of 8.6% with a diagnosis of malnutrition in 2019 appears to be lower than most published studies that assign diagnoses using clinical criteria. Estimates drawn from prior work vary greatly because they were conducted in disparate geographic locations, in different patient populations, over more than a 30‐year time period, and because they used varied criteria used for diagnosis.^3^, ^11^, ^12^, ^13^, ^14^, ^15^, ^16^ A recent systematic review of nutritional decline during hospitalization by Cass and Charlton^16^ included 15 studies (also across many countries) consisting of prospective cohorts, prospective cross‐sectional studies, one retrospective cohort study, and a mixed methods study that used a variety of clinical tools, including the AND/ASPEN criteria. Although the focus of this study was slightly different than ours and used clinical criteria rather than diagnosis codes to identify cases, the authors found 10%–65% of hospitalized patients experience nutritional decline during hospitalization, similar to prior estimates of the diagnosis. One trends study in children described increasing rates of undernutrition diagnoses in children from 2012 (4%) to 2019 (6%), which is similar to our findings.^17^

Our findings have important implications. The increase in diagnoses of malnutrition over time we observed is likely the result of several different factors, including an increased focus on improving identification of at‐risk patients^18^, ^19^; efforts to detect and treat malnutrition to improve clinical outcomes; efforts to improve coding of comorbid conditions or complications^20^; or increasing severity of illness of hospitalized patients.^21^, ^22^ If the main reason for the increase in prevalence we observed was increasing severity of illness, however, we would expect increasing use (or no change) of mechanical ventilation and an increase in deaths over time. The reduction in mortality between 2016 and 2019 among patients with a diagnosis of severe malnutrition suggests documentation and coding practices are also contributing to the change. Since coding is changing over time, studies of diagnostic codes or other retrospective data may not be able to answer the question of how many hospitalized patients experience clinically important malnutrition (i.e., malnutrition with phenotypic manifestations or downstream disease consequences).

The subjective nature of various diagnostic criteria may contribute to the variation in prevalence of malnutrition we have observed between this and prior studies. The AND/ASPEN criteria (Supporting Information S2: Table 1) do not require objective data, such as calorie counts, but instead use descriptors such as “poor enteral intake.” Weight loss is included, but the thresholds may be within the realms of normal weight fluctuation and do not clarify baseline weight (e.g., dry weight; last known weight; mean weight over last year).^23^, ^24^, ^25^ AND/ASPEN criteria have been validated against another subjective measure, the Subjective Global Assessment (SGA), but SGA was validated 40 years ago and uses clinician judgment of nutritional status taken from routine data collection during the history and physical exam–its relevance today is unclear.^26^, ^27^ Studies attempting to validate the AND/ASPEN or GLIM criteria against SGA are generally single‐center and conducted outside the United States.^4^, ^28^, ^29^

The GLIM criteria (Supporting Information S3 and S4: Tables 2 and 3) may address some of the challenges associated with the AND/ASPEN criteria,^4^, ^30^, ^31^ but are prone to some of the same errors of subjectivity. GLIM includes measures such as “inflammation” and “food intake,” which are poorly defined, and the weight threshold for a base diagnosis of malnutrition may still be within the realm of normal weight fluctuation (5%).

The variability and subjectivity of these criteria seem to contribute to the opportunity to exploit financial incentives to diagnose malnutrition.^32^ A failure to clarify these criteria could lead to further increases in the assignment of the diagnosis of malnutrition without clear connection to clinical outcomes, and the diagnoses are likely to be used differentially by hospitals with greater resources directed at coding and billing.

To get clearer estimates of the prevalence of clinically important disease in the US hospital population, studies in contemporary cohorts are needed to clarify diagnostic criteria (e.g., “food intake” and “inflammation”), and should include validated body composition measuring techniques, such as those proposed by Blackburn.^33^ Additionally, there needs to be consideration for comorbid conditions that impact in‐hospital weight measurement (such as loading with intravenous fluids or diuresis). Other areas to clarify include identification of the best method for calculating baseline weight and which standard methods of measurement of malnutrition might be included in diagnostic criteria (e.g., C‐reactive protein [CRP],^34^ muscle mass loss, or anthropometric measurements). In a recent Delphi study, using CRP as an additional criterion for making the diagnosis of malnutrition was proposed^34^; however, there are myriad other reasons CRP may be elevated in inpatients. The authors of the SGA suggested hand‐grip strength should accompany nutritional surveys to obtain more clinically important estimates.^27^ Of note, prior studies have demonstrated that laboratory values of albumin or pre‐albumin are not specific for malnutrition, and their use has fallen out of favor.^27^

The criteria used to diagnose malnutrition are cumbersome to collect and can fail to clarify the diagnosis.^35^ Even if the existing criteria were validated in a contemporary population, there are many reasons hospitalized patients experience poor enteral intake that may not be directly related to nutritional state: loss of appetite, quality of hospital food, cultural differences, requirements to be “nothing by mouth” for procedures and tests, or other factors. The issue of assigning a diagnosis of malnutrition is already posing challenges for both hospitals and payers: a recent Office of the Inspector General audit of a random sample of Medicare beneficiaries reported that, based on supportive clinical documentation, 27 out of 200 reviewed cases correctly reported severe malnutrition, and 94% of the remaining 173 resulted in overpayment of $1 billion nationally.^36^ Hospitals relying on malnutrition diagnoses for reimbursement may face significant financial risk during future audits without a cohesive, robust set of reproducible diagnostic methods to support malnutrition diagnoses.

There is also a need to identify treatment options for malnutrition that improve major clinical outcomes in medically ill inpatients. Although the mainstay of therapy is to increase nutrition via either enteral (EN) (including oral supplementation) or parenteral (TPN) feeding, enteral tube placement or central line placement for TPN may expose patients to unnecessary invasive procedures and are associated with harm. A recent Cochrane review also did not find any evidence EN or TPN affected short‐term mortality or adverse events.^37^ Increasing food prescription may not benefit those patients who are unable to obtain or eat food. Nutritional supplements may not be considered a traditional medical need and may not be covered by insurance. Patients residing in a “food desert” or with low socioeconomic status may not be able to access food. Inability to consume increased nutrition due to underlying medical issues may obviate the effects of increasing prescribed nutrition. Moreover, some of the appetite changes triggering a malnutrition screening score may be physiologically normal and protective.^38^ Well‐designed clinical trials using stringent criteria for entry, consistent interventions, and specific patient populations are needed to better understand the treatment effects of supplemental nutrition. Postdischarge targeted intervention may improve outcomes patients with malnutrition,^39^ but some prior studies of postdischarge interventions have been criticized for methodological flaws.^40^, ^41^ Future study should examine the effect of post‐discharge nutritional clinics in the United States.

There are also important limitations to our study. The database did not contain any reviewable clinical data including lab results, notes, or other relevant information. We therefore cannot delineate how the diagnosis of malnutrition was made. We did not examine the use of TPN because it is rarely prescribed for general medical patients except in select indications. Because of this, it is unlikely lacking data on TPN would impact our findings. Moreover, our study demonstrated a decrease in use of PEG tubes in all groups, though less in severe malnutrition. This is likely a reflection of a secular trend in reduction in PEG tube use. Our sampling stops at 2019, which was the last year provided at the time this study was conducted. It is possible the trends changed in 2020; however, due to the beginning of COVID‐19 pandemic, hospitalization data will be difficult to interpret. The data released from 2021 to 2022 will need to be analyzed, when available, to corroborate the trends detected in our study. Finally, we lacked clinical information informing our understanding of severity of illness. Future study should include detailed information on severity of illness, interventions provided, degree of follow‐up, and overall outcomes.

Use of diagnostic codes for malnutrition in hospitalized patients increased from 2016 to 2019, while the inpatient death rate in the population with malnutrition appeared to decrease, suggesting a change in coding practices contributed (at least in part) to the changes we observed. If we aim to understand the true prevalence of malnutrition or improve outcomes of patients with this diagnosis, we need better clarity of diagnostic criteria, standardization of coding practices, a definition of clinically important malnutrition, identification of effective treatments, acknowledgment of social drivers of malnutrition, and robustly validated tools for inpatients.

## CONFLICT OF INTEREST STATEMENT

The authors declare no conflict of interest.

## Supporting information

Supporting Information

Supporting Information

Supporting Information

Supporting Information
