# Supplementary material for: Malnutrition in hospitalized adults in the United States, 2016–2019
Source: J Hosp Med. 2024 Jul 9;19(12):1113–21. doi: 10.1002/jhm.13456 (PMC11613653; doi:10.1002/jhm.13456)
Supplement: Supplementary file 1 — Supporting Information [file JHM-19-1113-s002.docx]

Appendix A: ICD-10 codes used for comorbidities and Gagne score calculation

| **Comorbidity** | **ICD-10 (Diagnosis and procedure codes)** |
| --- | --- |
| **Non-severe malnutrition** | E44.x, E46.x |
| **Severe malnutrition** | E40.x, E41.x, E42.x, E43.x, R64 |
| **PAD** | I70.x, I71.x, I73.x, I77.1, I79.0, K55.1, K55.8, K55.9 |
| **Dementia** | F00.x, F01.x, F02.x, F03.x, G30.x, G31.x, F05.1 |
| **Chronic Lung Disease** | J40.x, J41.x, J42.x, J43.x, J44.x, J45.x, J46.x, J47.x, J60.x, J61.x, J62.x, J63.x, J64.x, J65.x, J66.x, J67.x, J68.x, I27.8, I27.81, I27.82, I27.83, I27.89, I27.9, J70.1, J70.3 |
| **Mild Liver Disease** | B18.x, K73.x, K77.x, B15.9 , B16.1 , B16.9 , B17.10 , B17.2 , B17.8 , B17.9 , B18.1 , B18.2 , B18.8 , B18.9 , B19.10 , B19.20 , B19.21 , K70.0 , K70.1 , K70.10 , K70.2 , K70.9 , K71.0 , K71.2 , K71.3 , K71.4 , K71.5 , K71.50 , K71.6 , K71.8 , K71.9 , K75.2 , K75.3 , K75.4 , K75.8 , K75.81 , K75.89 , K75.9 , K76.0 , K76.1 , K76.2 , K76.3 , K76.4 , K76.8 , K76.89 , K76.9 |
| **Severe Liver Disease** | K72.x, K74.x, I85.x, B15.0 , B16.0 , B16.2 , B17.0 , B17.11 , B18.0 , B19.0 , B19.11 , B19.21 , B19.9 , K70.3 , K70.30 , K70.31 , K70.4 , K70.40 , K70.41 , K71.1 , K71.10 , K71.11 , K71.51 , K71.7 ,K76.5 , K76.6 , K76.7 , K76.81 , Z94.4 |
| **Diabetes without complications** | E08.9, E0.9, E10.9, E11.9, E12.9, E13.9, E14.9 |
| **Diabetes with complications** | E08.1-E08.8, E09.1-E09.8, E10.1-E10.8, E11.1-E11.8, E12.1-E12.8, E13.1-E13.8 |
| **Paralysis** | G81.x, G82.x, G83.x, G04.1, G11.4, G80.1, G80.2, G83.0, G83.1, G83.2, G83.3, G83.4, G83.9 |
| **Renal Failure** | N18.x, N19.x, I12.x, I13.x, N03.x, N04.x, N05.x, N07.x, Z49.x |
| **Metastatic Cancer** | C77.x, C78.x, C79.x, C80.x |
| **Malignancy** | C00.x, C01.x, C02.x, C03.x, C04.x, C05.x, C06.x, C07.x, C08.x, C09.x, C10.x, C11.x, C12.x, C13.x, C14.x, C15.x, C16.x, C17.x, C18.x, C19.x, C20.x, C21.x, C22.x, C23.x, C24.x, C25.x, C26.x, C30.x, C31.x, C32.x, C33.x, C34.x, C37.x, C38.x, C39.x, C40.x, C41.x, C43.x, C45.x, C46.x, C47.x, C48.x, C49.x, C50.x, C51.x, C52.x, C53.x, C54.x, C55.x, C56.x, C57.x, C58.x, C60.x, C61.x, C62.x, C63.x, C64.x, C65.x, C66.x, C67.x, C68.x, C69.x, C70.x, C71.x, C72.x, C73.x, C74.x, C75.x, C76.x, C81.x, C82.x, C83.x, C84.x, C85.x, C88.x, C90.x, C91.x, C92.x, C93.x, C94.x, C95.x, C96.x, C97 |
| **HIV** | B20.x, B21.x, B22.x, B24.x, Z21.x, O98.7, O98.71, O98.711, O98.712, O98.713, O98.72, O98.73 |
| **Lymphoma** | C81.x, C82.x, C83.x, C84.x, C85.x, C86.x, C88.x, C90.x, C96.x |
| **Asthma** | J45.x |
| **COPD** | J42.x, J43.x, J44.x |
| **CHF** | I42.x, I43.x, I50.x, I09.81, I11.0, I13.0, I13.2, I25.5, I42.0, I42.1, I 42.5, I42.6, I42.7, I42.8, I42.9, P29.0 |
| **Weight Loss** | E40.x, E41.x, E42.x, E43.x, E44.x, E46.x, R64.x, R63.4, R63.6, R62.7 |
| **Obesity** | E65.x, E66.x, E66.0, E66.01, E66.1, E66.2, E66.8, E66.9, R93.9, Z68.3, Z68.30, Z68.31, Z68.32, Z68.33, Z68.34, Z68.35, Z68.36, Z68.37, Z68.38, Z68.39, Z68.4, Z68.41, Z68.42, Z68.43, Z68.44, Z68.45 |
| **Alcohol Abuse** | T51.x, F10.x, K70.x, G62.1, I42.6, K29.2, K29.20, K29.21, K70.0, K70.3, K70.9, P04.3, Q86.0, Z50.2, Z65.8, Z71.4, Z71.41, Z71.42, Z72.1 |
| **Arrhythmia** | I44.x, I45.x, I47.x, I48.x, I49.x, I44.1, I44.2, I44.3, I45.6, K45.9, R00.0, R00.1, R00.8, R00.9, T82.1, T82.11, T82.110, T82.110A, T82.110D, T82.110S, T82.111, T82.111A, T82.111D, T82.111S, T82.118, T82.118A, T82.118D, T82.118S, T82.119, T82.119A, T82.119D, T82.119S, T82.12, T82.120, T82.120A, T82.120D, T82.120S, T82.121, T82.121A, T82.121D, T82.121S, T82.128, T82.128A, T82.128D, T82.128S, T82.129, T82.129A, T82.129D, T82.129S, T82.19, T82.190, T82.190A, T82.190D, T82.190S, T82.191, T82.191A, T82.191D, T82.191S, T82.198, T82.198A, T82.198D, T82.198S, T82.199, T82.199A, T82.199D, T82.199S, Z45.0, Z45.01, Z45.0101, Z45.018, Z45.02, Z45.09, Z95.0, Z95.810 |
| **Coagulopathy** | D65.x, D66.x, D67.x, D68.x, D69.x, D69.1, D69.3, D69.4, D69.5, D69.6, R23.3, D75.82, D75.89 |
| **Fluid Electrolyte Disorder** | E86.x, E87.x, E22.2 |
| **Deficiency Anemia** | D51.x, D52.x, D53.x, D50.1, D50.8, D50.9 |
| **Psychoses** | F20.x, F21.x, F22.x, F23.x, F24.x, F25.x, F28.x, F29.x, F06.0, F06.2, F30.2, F31.2, F31.5, F31.64, F32.3, F33.3 |
| **Pulmonary Circulatory Disorders** | I26.x, I27.x, I28.x, I28.0, I28.8, I28.9 |
| **Hypertension Complicated** | I11.x, I12.x, I13.x, I15.x |
| **Hypertension Uncomplicated** | I10.x |
| **Mechanical Ventilation** | 5A1935Z, 5A1945Z, 5A1955Z |
