# Supplementary material for: Malnutrition in hospitalized adults in the United States, 2016–2019
Source: J Hosp Med. 2024 Jul 9;19(12):1113–21. doi: 10.1002/jhm.13456 (PMC11613653; doi:10.1002/jhm.13456)
Supplement: Supplementary file 2 — Supporting Information [file JHM-19-1113-s004.docx]

**Supplementary Table 1**: ASPEN Criteria, Adapted from original ASPEN guidelines

|  | **Acute Illness or Injury** | | **Chronic*** Illness** | | **Social/Environmental Circumstances** | |
| --- | --- | --- | --- | --- | --- | --- |
|  | **Non-severe** | **Severe** | **Non-severe** | **Severe** | **Non-severe** | **Severe** |
| **Energy Intake^a^** | < 75% of needs for > 7 days | ≤ 50% of needs for ≥ 5 days | < 75% of needs for ≥ 1 month | ≤ 75% of needs for ≥ 1 month | < 75% of needs for ≥ 3 months | ≤ 50% of needs for ≥ 1 month |
| **Weight loss^b^** | 1-2% over 1 week | >2% over 1 week | 5% over 1 month | >5% over 1 month | 5% over 1 month | >5% over 1 month |
|  | 5% over 1 month | >5% over 1 month | 7.5% over 3 months | >7.5% over 3 months | 7.5% over 3 months | >7.5% over 3 months |
|  | 7.5% over 3 months | >7.5% over 3 months | 10% over 6 months | >10% over 6 months | 10% over 6 months | >10% over 6 months |
|  |  |  | 2% over 1 year | >20% over 1 year | 2% over 1 year | >20% over 1 year |
| **Physical Findings** |  |  |  |  |  |  |
| **Body fat loss^c^** | Mild | Moderate | Mild | Severe | Mild | Severe |
| **Muscle mass loss (wasting of temples, clavicles, shoulders, interosseus muscles, scapula, calf)^d^** | Mild | Moderate | Mild | Severe | Mild | Severe |
| **Fluid accumulation ^e^** | Mild | Moderate - Severe | Mild | Severe | Mild | Severe |
| **Reduced grip strength^f^** | NA | Measurably reduced | NA | Measurably reduced | NA | Measurably reduced |
| ^a^Relative to estimated intake requirements  ^b^Weight change over time reported as a percentage of weight lost from baseline  ^c^Loss of subcutaneous fat (orbital, triceps, overlying ribs, etc.)  ^d^Loss of muscle mass (temporalis, pectoralis, deltoids, interosseus muscles, latissimus, trapezius, quadriceps, gastrocnemius)  ^e^Fluid retention may result in weight gain  ^f^Relative to normative standards supplied by device manufacturer  *Need 2/6 characteristics to diagnose severe or non-severe malnutrition  **Height/weight should be measured  ***Chronic is a disease or condition that lasts 3 months or longer | | | | | | |
