# Supplementary material for: Malnutrition in hospitalized adults in the United States, 2016–2019
Source: J Hosp Med. 2024 Jul 9;19(12):1113–21. doi: 10.1002/jhm.13456 (PMC11613653; doi:10.1002/jhm.13456)
Supplement: Supplementary file 3 — Supporting Information [file JHM-19-1113-s003.docx]

**Supplementary Table 2**: GLIM malnutrition Criteria, adapted from original GLIM guidelines

| **Phenotypic Criteria^*^** |  |  | **Etiologic Criteria*** |  |
| --- | --- | --- | --- | --- |
| **Weight Loss** | **Low BMI (kg/m^2^)** | **Reduced muscle mass^a^** | **Reduced food intake or assimilation^b^** | **Inflammation^c^** |
| >5% if < 6 months | < 20 if < 70 years | Reduced by validated techniques | ≤50% of needs for more than 1 week | Acute disease/injury |
| >10% if ≥ 6 months | < 22 if > 70 years |  | Any reduction >2 weeks | Chronic disease related |
|  | Asia: < 18.5 if < 70 years |  | Any chronic GI condition that impacts absorption |  |
|  | Asia: < 20 if ≥ 70 years |  |  |  |
| *Need 1 phenotypic and 1 etiologic criterion to diagnose malnutrition  ^a^Fat-free mass index by dual-energy absorptiometry, bioelectrical impedance analysis, CT, or MRI. If not available, standard anthropometric measures like mid-arm muscle or calf circumference may be used. Need to adapt to race. Functional assessments (hand-grip) may be supportive.  ^b^GI symptoms such as dysphagia, nausea, vomiting, diarrhea, constipation, abdominal pain. Need to note symptom intensity, frequency, duration. Assimilation may be indicated by malabsorptive disorders (short gut, pancreatic disease, bariatric surgery) or other nutritional-intake reducing disorders (strictures gastroparesis, malabsorption, etc).  ^c^Acute disease related (e.g., infection, burns, trauma, head injury); chronic disease related (malignancy, COPD, CHF, renal disease, any disease with chronic or recurrent inflammation). Severe inflammation not generally associated with chronic disease. Can use CRP as supportive measure. | | | | |
