# Supplementary material for: Malnutrition in hospitalized adults in the United States, 2016–2019
Source: J Hosp Med. 2024 Jul 9;19(12):1113–21. doi: 10.1002/jhm.13456 (PMC11613653; doi:10.1002/jhm.13456)
Supplement: Supplementary file 4 — Supporting Information [file JHM-19-1113-s001.docx]

**Supplementary Table 3**: Severity of malnutrition by GLIM, adapted from original GLIM guidelines.

|  | **Phenotypic Criteria** | | |
| --- | --- | --- | --- |
| **Stage 1 / Moderate^a^** | **Weight loss** | **Low BMI (kg/m^2^)** | **Reduced Muscle Mass^c^** |
|  | 5-10% within past 6 months | < 20 if < 70 years | Mild to moderate deficit |
|  | 10-20% beyond 6 months | < 22 if ≥ 70 years |  |
| **Stage 2 / Severe^b^** | >10% within the past 6 months | < 18.5 if < 70 years | Severe deficit |
|  | >20% beyond 6 months | < 20 if ≥ 70 years |  |
| ^a^Requires 1 phenotypic criterion to meet this grade  ^b^Requires 1 phenotypic criterion to meet this grade  ^c^Appendicular lean mass index by dual-energy absorptiometry, bioelectrical impedance analysis (BIA), CT or MRI. If not available, standard anthropometric measures like mid-arm muscle or calf circumference may be used. | | | |
